# Paris Hospitals

**Published:** 1858-05

**Authors:** 


*Paris Hospitals.*—The care of the sick and the alleviation of their sufferings are duties of which no great society can be unmindful. Poverty and disease always work beneath the skirts of prosperity in large cities. Humanity suggests attention to their claims; security demands it, if only in the interests of public health. We have lately afforded an appreciation of the numerous institutions, the liberal funds, and the devoted services, which private generosity places at the disposal of the pauper sick of London. Documents recently published afford the means of a like analysis of the charitable resources of Paris. This we shall attempt, from time to time. Sixteen hospitals permit the reception of 7,000 diseased persons at the same time in that city, while they employ the energies of 67 physicians and 38 surgeons, the most illustrious members of the medical body. Fifteen dispensers, 179 house surgeons, and an army of dressers, are here engaged in the same sacred duties with their seniors. In addition to these, there are employed in these hospitals 22 chaplains, 774 nurses, and 409 general servants. The annual expense amounts to 5,500,000 francs, and more than 100,000 patients are annually admitted. The impressive vastness of these figures enforces thought. The splendid munificence of such a system must be admired. The mercy which makes so vast a concession to the cure of the wounded is of a quality not strained :

> "It droppeth as the gentle rain from heaven
> Upon the place beneath : it is twice blessed—
> It blesseth him that gives and him that takes."

The devotion of the most illustrious of our brethren to this holy labor earns for the class whom they represent a proud title to esteem, whilst it brings with it the enviable consciousness of a high duty well performed. Such charity is nobility's true badge. But the philanthropist is here also a patriot. No one can say how much of the longevity in great cities is due to the separation of the sick from the healthy. A little leaven leaveneth the whole. An infinitely smaller number of sick may well become centres of wide spreading circles of disease. The state, too, suffers, wherever disease becomes endemic, or when its ravages are feebly or unskillfully strayed. The deterioration of race—the degeneracy of physical power—the enfeeblement of body, at least, which necessarily result, inflict an economic loss. Every case of preventible disease presents a public loss. So also does each case of neglected illness or prolonged convalescence in the laboring man ; for

> "Infirmity doth still neglect all office
> Whereto our health is bound."

Thus medicine claims a position in the calculations of the political economist, and the physician rises to the highest rank amongst practical statesmen.—*London Lancet— Virginia Medical Journal.*

